# Large-scale monitoring of effects of clothianidin-dressed oilseed rape seeds on pollinating insects in northern Germany: residues of clothianidin in pollen, nectar and honey

**DOI:** 10.1007/s10646-016-1723-x

**Published:** 2016-09-20

**Authors:** Daniel Rolke, Markus Persigehl, Britta Peters, Guido Sterk, Wolfgang Blenau

**Affiliations:** 1 Institut für Bienenkunde (Polytechnische Gesellschaft), Goethe University Frankfurt, Oberursel, Germany; 2 tier3 solutions GmbH, Leverkusen, Germany; 3 IPM Impact, Kuringen, Belgium

**Keywords:** Honey bees, Bumble bees, Mason bees, Neonicotinoids, Residues, Seed treatment

## Abstract

This study was part of a large-scale monitoring project to assess the possible effects of Elado^®^ (10 g clothianidin & 2 g β-cyfluthrin/kg seed)-dressed oilseed rape seeds on different pollinators in Northern Germany. Firstly, residues of clothianidin and its active metabolites thiazolylnitroguanidine and thiazolylmethylurea were measured in nectar and pollen from Elado^®^-dressed (test site, T) and undressed (reference site, R) oilseed rape collected by honey bees confined within tunnel tents. Clothianidin and its metabolites could not be detected or quantified in samples from R fields. Clothianidin concentrations in samples from T fields were 1.3 ± 0.9 μg/kg and 1.7 ± 0.9 μg/kg in nectar and pollen, respectively. Secondly, pollen and nectar for residue analyses were sampled from free flying honey bees, bumble bees and mason bees, placed at six study locations each in the R and T sites at the start of oilseed rape flowering. Honey samples were analysed from all honey bee colonies at the end of oilseed rape flowering. Neither clothianidin nor its metabolites were detectable or quantifiable in R site samples. Clothianidin concentrations in samples from the T site were below the limit of quantification (LOQ, 1.0 µg/kg) in most pollen and nectar samples collected by bees and 1.4 ± 0.5 µg/kg in honey taken from honey bee colonies. In summary, the study provides reliable semi-field and field data of clothianidin residues in nectar and pollen collected by different bee species in oilseed rape fields under common agricultural conditions.

## Introduction

All bee species rely on pollen and nectar as food sources. In collecting these plant substrates, they serve as economically valuable pollinators of cultivated crop plants and contribute to ecosystem services (Klein et al. 2007). Not only honey bees (*Apis* spp.) but also bumble bees (*Bombus* spp.) and mason bees (*Osmia* spp.) are commercially reared for pollination services and can be more or less ifically targeted to crop monocultures. In addition, various species of bumble bees and mason bees (among many other bee species) are common and widespread in the wild and, to some extent, can also be attracted by crop monocultures. Since agricultural crop plants are commonly treated with plant protection products (PPPs) against insect pests, pollinating insects such as bees may potentially be affected by this treatment. Thus, PPPs contribute to the multiple and varying stressors bees are exposed to and which also include habitat loss, agricultural intensification, parasites and pathogens (Potts et al. 2010). In particular, systemically acting PPPs of the neonicotinoid class of compounds are often held responsible for losses of honey bee colonies and declines in the abundance of wild bees (Sánchez-Bayo, 2014; Goulson et al. 2015; Pisa et al. 2015, Rundlöf et al. 2015). As synthetic nicotine analogues, neonicotinoids affect the nicotinic acetylcholine receptor in the insect brain (for reviews, see: Tomizawa and Casida 2005; Jeschke et al. 2013). Neuroactive neonicotinoids are commonly used as seed dressings in a variety of crops including oilseed rape (OSR). The growing plant absorbs the insecticide, which is distributed to all plant tissues and substrates, including pollen and nectar (Elbert et al. 2008). Because of this systemic activity, neonicotinoids can be applied as a seed dressing or to soil at low rates, which reduces the need for foliar insecticide applications that are applied at much greater rates.

The European Union has temporarily suspended the use of three neonicotinoids (clothianidin, imidacloprid and thiamethoxam) for seed treatment, soil application and foliar treatment in bee attractive crops (European Commission 2013) to allow for in depth studies of their environmental effects. Many laboratory and semi-field studies have provided data about lethal and sublethal effects of neonicotinoids to bees under certain application regimes and at specific concentrations (Godfray et al. 2014, Godfray et al. 2015). However, from an environmental perspective they have been criticised (Cresswell and Thompson 2012; Guez 2013; Carreck and Ratnieks 2014; Godfray et al. 2014, Godfray et al. 2015), e.g., for not using field realistic doses or for subjecting bees exclusively to food spiked with neonicotinoids under laboratory conditions. Because of the basic toxicological principle of the relationship between dose and response, a central question is whether concentrations of neonicotinoid residues in pollen and nectar reach levels that are deleterious to bees under common agricultural practice and landscape conditions.

In 2013, a comprehensive monitoring project was initiated to examine potential effects of clothianidin seed dressing on pollinators under common agricultural practice. This large-scale field study aimed to investigate possible side effects of clothianidin-dressed OSR seeds at the landscape level on various pollinators under actual agricultural conditions (Schmuck and Lewis 2016). This project consisted of four different pollinator studies performed in the project area at the same time: a honey bee monitoring study (Rolke et al. 2016), a mason bee monitoring study (Peters et al. 2016), a bumble bee monitoring study (Sterk et al. 2016), and a residue analysis of pollen and nectar from foraging honey bees in tunnel tents placed over the test crop as well as of pollen, nectar and honey collected by free flying honey bees, bumble bees and mason bees (present study). The studies were conducted in Mecklenburg-West Pomerania (Mecklenburg-Vorpommern, Germany) at two circular study sites of approximately 65 km^2^. Each site surrounded the investigated bee hives and nesting shelters. In autumn 2013, study fields were either drilled with clothianidin-free OSR seeds (reference site, R) or with clothianidin-dressed OSR seeds (Elado^®^: 10 g clothianidin and 2 g β-cyfluthrin/kg seed (test site, T)).

The aim of the study part presented here was to provide reliable data on residue concentrations of clothianidin and its active metabolites thiazolylmethylurea (TZMU) and thiazolylnitroguanidine (TZNG) in OSR pollen, nectar and honey produced under common agricultural practice. Accordingly, honey bee and bumble bee hives as well as mason bee nesting blocks were installed at study locations within the reference and test sites. For one approach, bees were allowed to forage freely within their natural flight radius. Pollen collected by all three bee species was sampled and analysed. In addition, nectar collected by honey bee workers and, at the end of OSR flowering, honey from all honey bee colonies were sampled. Data from this field realistic scenario was then compared to data obtained from a worst-case scenario, in which honey bees were forced to forage on OSR only. This was achieved by using a semi-field tunnel tent approach.

The results of this study part serve as basic data for the understanding and interpretation of the results obtained in the bee monitoring studies (Rolke et al. 2016; Sterk et al. 2016; Peters et al. 2016) as well as providing the necessary background for a general discussion on field realistic exposure of bees to clothianidin and its metabolites.

## Material and methods

### Description of the study fields, study field subareas and study locations

The study was conducted at two neighbouring study sites in the vicinity of Sternberg, northern Germany. Each study site covered an area of approximately 65 km² with a diameter of 9 km. Both sites together provided in total nearly 1,800 ha of OSR crops (27 % of arable land). During OSR flowering, no other bee attractive crops were present. The selection of the project area and the study sites took place in summer 2013 and is described in detail in Heimbach et al. (2016). Before drilling, soil samples were collected from all study fields for the analysis of clothianidin residues and soil characterisation. In addition, clothianidin loadings of the OSR seeds were analysed and the entire development of OSR from drilling to harvest was monitored. The results of these analyses are reported in Heimbach et al. (2016). Farmers cultivated OSR and other crops at both study sites according to their common procedures and at their own discretion. They were allowed to compensate for the missing insecticidal dressing in OSR fields at the R site as necessary by foliar spray applications of pyrethroids according to their own selection. Except for the clothianidin dressing of OSR seeds at the T site, no further neonicotinoid was used from autumn 2013 until summer 2014 at the study fields (for details of farming and PPP applications in the study area, see: Heimbach et al. (2016).

**Fig. 1:**
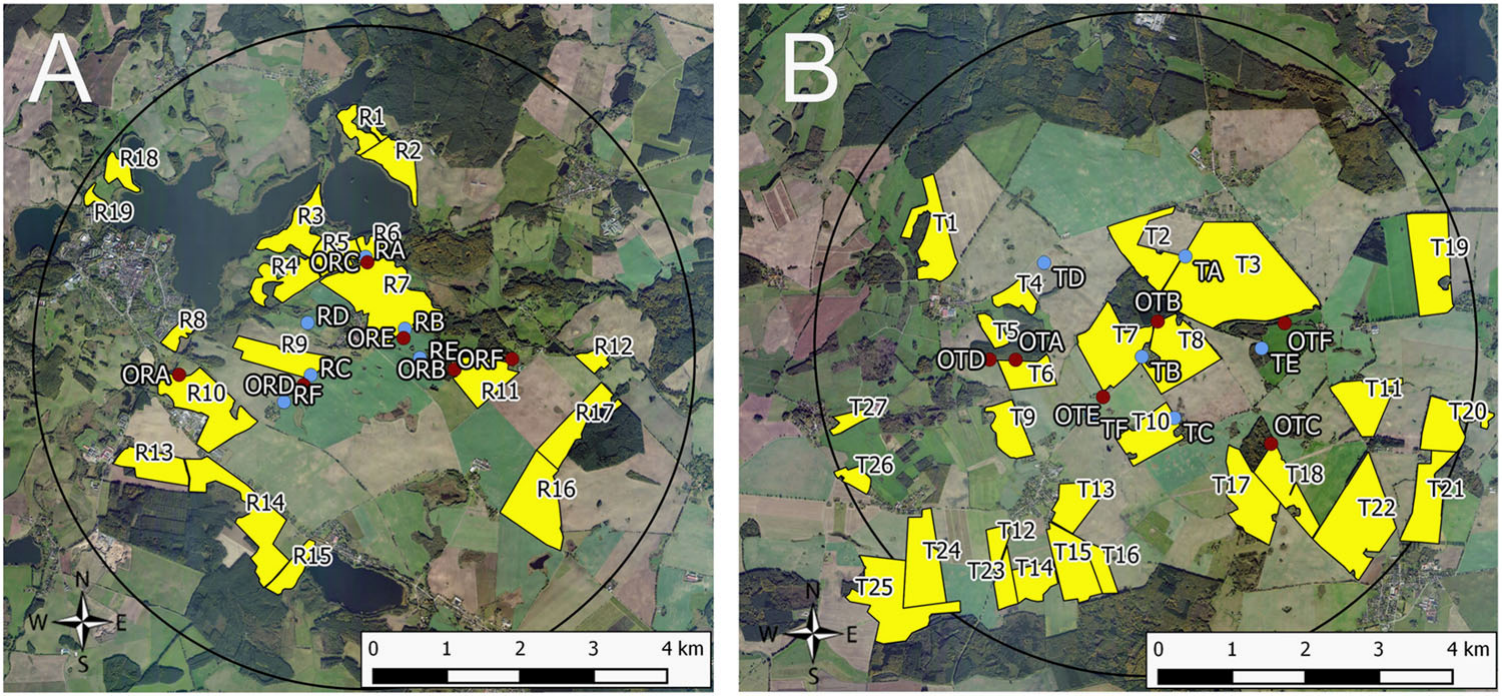
Study locations and study fields at the reference site (**a**) and test site (**b**). Study locations of honey bees and bumble bees are indicated by blue points. Study locations of mason bees are indicated by red points. Yellow polygons indicate OSR study fields. Circle diameter = 9 km

**Fig. 2:**
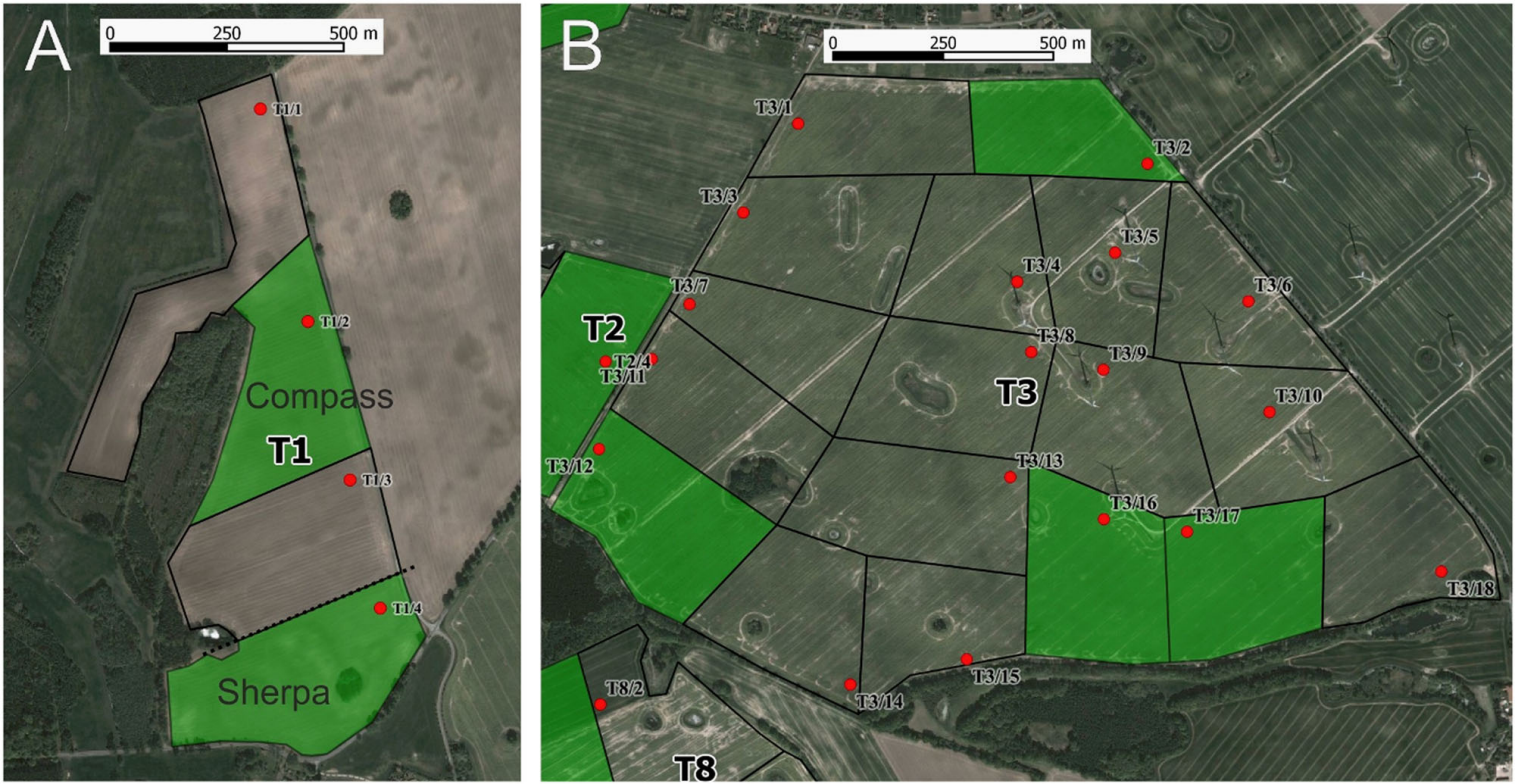
Example of subdivision of study fields into subareas. **a** Study field T1 with four subareas and corresponding tunnel tent locations (red dots; the *broken line* indicates the border between the two OSR varieties, three tunnel tent locations were placed in OSR variety “Compass” and one subarea covers the variety “Sherpa”). **b** Study field T3 (198 ha, divided into 18 subareas). For (**a**) and (**b**), only samples of subareas which are indicated by green filling have been taken

In autumn 2013, Elado^®^ (10 g clothianidin & 2 g β-cyfluthrin/kg seed)-dressed OSR seeds were drilled at all 18 study fields (total area approximately 792 ha) at the T site, whereas Elado^®^-free OSR seeds were drilled at all 17 study fields (total area approximately 615 ha) at the R site (Fig. 1). The median area of study fields was 35.3 ha (1.22–198.0 ha) at the T site and 33.5 ha (9.0–97.3 ha) at the R site. On average, 3.4 ± 1.1 kg/ha of OSR seeds were drilled in the study fields. 1 kg of OSR seeds from the T site contained on average 8.0 ± 1.2 g clothianidin, which amounts to 28.8 ± 10.0 g/ha when the seeds were drilled in the T fields, and this in turn amounts to 19.2 ± 6.7 μg clothianidin per kg soil in the uppermost 10 cm of the soil after drilling assuming an equal distribution of clothianidin in the top 10 cm soil and a soil density of 1.5 kg/l. OSR seeds in the R fields exhibited a median loading of 0.02 g clothianidin per kg seeds. In the R site, the resulting amount of clothianidin per unit area was 0.19 ± 0.25 g/ha that was 0.7 % of the analysed concentration of the T site. For a detailed description of the seed treatment, OSR fields and planting, see Heimbach et al. (2016). Each study field was divided into equally sized sampling plots, called subareas, of approximately 10 ha by means of the GIS programme QGIS. The main aim of subdividing the study fields into subareas was to achieve an appropriate number of samples related to the field size. Examples of the subdivision of two study fields are shown in Fig. 2. Study field T13 was an OSR variety demonstration field and each of the 22 OSR varieties was represented by a subarea, which was smaller than 1 ha. In accordance to the given criteria, the study fields of the R site were subdivided into 58 subareas, and the 18 study fields of the T site were divided into 96 subareas.

For free flying bees, six study locations were selected each at the R and T sites (Fig. 1). Three out of the six study locations per study site were established at the edge of an OSR field, whereas the other three were situated 400 m (for honey bees and bumble bees) or 100 m (for mason bees) distant from the nearest OSR field. Honey bees and bumble bees were placed at the same study locations, whereas mason bees were positioned at different locations taking into account their maximum flight distances. For detailed descriptions of the study locations, see Rolke et al. (2016), Sterk et al. (2016), and Peters et al. (2016).

### Estimation of OSR plant density at tunnel tent positions

The density of emerged OSR plants was estimated before stem elongation in March 2014 to compare between the densities of drilled seeds (data provided by Heimbach et al. 2016) and developed plants at the later tunnel tent positions. At each position, a frame of 1 × 1 m was randomly placed 10 times on the ground within a radius of 5 m. The number of emerged OSR plants was counted within the frame. Mean numbers of OSR plants were calculated for each tunnel tent position.

### Tunnel tents and tunnel tent arrangement

Tunnel tent experiments were conducted to sample nectar and pollen from OSR plants in the study fields via honey bees under semi-field conditions. The tunnel tents (10 × 5 × 2.5 m) used in this study were semi-circular in cross-section and constructed out of a tubular steel frame, covered with synthetic gauze (mesh size ca. 2 mm). The tunnel tents had a sampling area of approximately 50 m² (at least 45 m² covered with OSR) and were placed in subareas of the study fields for 3 days in a north-south direction, with a minimum distance of 20 m to the edge of the OSR field (Supplementary Figure S1). To ensure that samples of nectar and pollen originated from OSR flowers of the study field subarea, honey bees were enclosed in the tunnel tents at least 1 day before sampling. Each honey bee colony (see 2.4) was used only once and was removed after successfully collecting pollen and nectar.

### Preparation and management of bees

#### Honey bee colonies

Honey bee colonies, *Apis mellifera carnica* (Apidae), bred according to normal beekeeping practice, disease-free and queen-right were used. All queens were offspring (F1) of the same mother queen. Two different types of honey bee hives were used in this study. For semi-field tunnel tent experiments, small hives (“Mini Plus”, 30 × 30 × 30 cm, 6 combs, and approximately 2,500 bees) were used. These hives were placed inside the tunnel tents at the northwest corner with south-facing entrances.

Hives for free flying honey bees were larger in size and consisted of one brood chamber and 1–2 honey supers (“Zander”, measures of one level (brood chamber and honey supers): 50 × 43.5 × 23 cm, 10 combs per level). Eight hives were placed with south-facing entrances at each of the 12 study locations at the beginning of OSR full flowering on 22/23 April 2014. Rolke et al. (2016) give a detailed description of the preparation, placement and management of the honey bee colonies.

#### Bumble bee colonies

The buff-tailed or large earth bumble bee, *Bombus terrestris dalmatinus* (Apidae), was used, as this subspecies is commercially available and widely used for pollination indoors and outdoors. At each study location, one single hive was established for the sampling of returning bumble bees loaded with pollen on 25 April 2014 (in addition to 9 hives established for assessment of colony development, see, Sterk et al. 2016). All hives were positioned south-facing to be protected against wind and rain at the start of OSR flowering. They were set up on top of concrete blocks about 30 cm above the ground. To achieve comparability, every colony contained a mother queen from the same hibernation batch. A detailed description of the preparation, placement and management of the bumble bee colonies can be found in Sterk et al. (2016).

#### Mason bees

In total, 18,000 cocoons of the red mason bee, *Osmia bicornis* (Megachilidae), were used in this study. The cocoons were kept at −2 °C to +4 °C over the winter period 2013/2014. Before the cocoons were placed in the study area, they were incubated for 5 days at 8 °C followed by 2 days at 11 °C. At each study location, two cardboard boxes with 750 cocoons each were placed into nesting shelters at the start of OSR flowering on 21 April 2014. Nesting shelters were positioned south-east-facing so that they were exposed to direct sunlight but protected against rain. In all cases, the nesting shelters were placed in front of a forest, a hedge or large shrubs to ensure similar protection from wind. Inside the nesting shelters, nesting blocks composed of 20 medium-density fibreboards (16 × 16 cm) were provided. Each board contained 10 rows of nesting holes arranged in parallel and each row contained 10 nesting holes (8 mm in diameter). See Peters et al. (2016) for a more detailed description of preparation and placement of the mason bees.

### Sample collection

#### Pollen collection

In the tunnel tents, sampling started on 23 April 2014 and lasted until 19 May 2014. On 21/22 April, all OSR fields appeared to be in full flower and by 22 May 2014 the flowers in the majority of study fields had withered (Heimbach et al. 2016). During these 4 weeks, samples from 34 out of 58 subareas in the R site and 41 out of 96 subareas in the T site were collected, with collection of at least one sample per study field. The colonies were set up inside the tunnel tents 2 days before sampling and bees were allowed to fly and habituate to the test conditions. For the collection of pollen samples, pollen traps (a punched plate through which pollen-carrying bees must crawl to separate the pollen pellets from the bees’ legs and a fine meshed grid to store these pellets) were attached in front of the entrance of the honey bee hives the evening before sampling. One sample with a minimum amount of 100 mg pollen was collected once per tent location until noon of the sampling day.

Colonies of free flying honey bees were placed at the study locations on 21/22 April 2014, with 21 April 2014 set as day after placement (DAP) 0. Pollen samples were taken twice from all 96 colonies (8 colonies per study location) at two different time points during OSR flowering, on DAP 15 and DAP 19/23. Pollen traps (a grid through which pollen-carrying bees must crawl to separate the pollen pellets from the bees’ legs and a fine meshed grid to store these pellets) were introduced into the bottom board of the hives the day before sampling. On the following day (sampling day), pollen traps were removed and the entire pollen yield was transferred to bottles and shaken in order to mix the pollen properly. Out of this mixture, subsamples of a minimum of 300 mg were taken.

Pollen samples from bumble bees were taken once at every study location during OSR flowering (DAP 19). At each bumble bee hive, 11–21 returning workers with pollen loads were caught and immediately transferred to dry ice. At least 230 mg of pollen was collected from the legs of the bumble bees.

Pollen samples from mason bees were taken once at every study location during OSR flowering (DAP 23). Accordingly, the nesting blocks were opened and 10 subsamples were collected from the rear end of an active, provisioned nesting cell by retrieving the pollen with a micro spoon and combined in a pooled sample with a minimum of 200 mg pollen.

All pollen samples were transferred separately into 15 ml tubes and stored frozen (between −10 °C and −43 °C) until analysis.

#### Nectar collection

For practical reasons, nectar samples were taken from honey bees only, both in tunnel tents and from free flying individuals. About 200 returning honey bee foragers per sample were caught at the hive entrances by using a vacuum collector and immediately transferred to dry ice and stored frozen (between −10 °C and −43 °C) until dissection. The honey stomachs of the collected honey bees were dissected in the laboratory (according to Carreck et al. 2013) and the nectar (minimum of 200 mg) was discharged into a 15 ml tube. All nectar samples were stored frozen (between −10 °C and −43 °C) until analysis.

#### Honey collection

Honey was only harvested from colonies of free flying honey bees. The harvest of spring honey took place immediately after the end of OSR flowering (DAP 32). Combs from individual colonies were uncapped and honey from each colony was extracted separately by the use of a radial extractor 42000 Voll (Carl Fritz Imkereitechnik Mellrichstadt, Germany). Honey samples (minimum 5 g) were transferred into 15 ml tubes and stored frozen (between −10 °C and −43 °C) until analysis.

### Residue analysis

Residue analyses were performed by Eurofins Agroscience Services Chem GmbH (Hamburg, Germany). The analytical method was based on the multi-residue sample preparation technique QuEChERS (“Quick Easy Cheap Effective Rugged Safe”, see Lehotay 2006) according to the European Standard EN15662:2008 (2009). Around 100 mg each of a homogenised specimen of pollen, nectar or honey was weighed into a 50 ml centrifuge tube. The exact weight of each sample was documented and used for calculating the residue concentration. To adjust the water content, 10 ml of water was added. For extraction, 10 ml acetonitrile was added to each tube. The centrifuge tube was capped and shaken by hand for at least 2 min. Thereafter, 4.0 g of MgSO_4_, 1.0 g of NaCl, 1.0 g of trisodium citrate dihydrate and 0.5 g of disodium hydrogen citrate sesquihydrate were added. The centrifuge tube was capped again and immediately shaken by hand for ≥1 min. The sample tube was centrifuged for 4 min at 4,000 rpm. Thereafter, an aliquot of exactly 6 ml of the acetonitrile phase was transferred to a 15-ml centrifuge tube and evaporated to dryness using a nitrogen stream and water bath at 40 °C. The residue was carefully taken up in exactly 1 ml of mixed internal standard solution (water/acetonitrile/formic acid, 75/25/0.1 v/v/v, final concentration: 0.3 ng/ml). The final solvent was equal to the solvent used for preparation of solvent-based standard solutions. If necessary, the uptake was supported by vortex and/or ultrasonic bath, and brief centrifugation was carried out afterwards to separate insoluble particles. Extracts were transferred to HPLC vials for analysis. Calibration solutions were prepared in mixed internal standard solutions ranging from 0.015 ng/ml – 2.0 ng/ml.

For clothianidin and its metabolites TZNG and TZMU, the quantification was performed by internal standardisation using stable-labelled internal standards in pure solvent. A calibration curve was established with at least six concentration levels and used for quantification. For each calibration curve, the coefficient of determination *R*² was >0.979. The chromatographic system used for the determination of clothianidin and its metabolites TZNG and TZMU was a high performance liquid chromatograph with reversed phase chromatography (Zorbax RRHD Eclipse Plus C18, 50 × 2.1 mm, 1.8 µm column) coupled with tandem mass spectrometry (MS/MS) with electrospray ionisation (AB Sciex API 6500 Triple Quadruple Mass Spectrometer, Analyst version 1.6.2). The limit of quantification (LOQ) was 1.0 µg/kg and the limit of detection (LOD) 0.3 µg/kg for all three compounds.

### Data analysis

Average residue concentrations were calculated using 0.0 µg/kg for individual concentrations “<LOD” and 0.65 µg/kg (= mean value for range between LOD and LOQ) for concentrations “<LOQ” to account for the fact that residues were detected and provide a reasonable estimate regarding potential biological effects. No separate average concentrations were calculated in cases where a minimum of 90 % of values were < LOD or < LOQ. The results are presented both as mean ± standard deviation (SD) as well as median values. The mean plant densities were compared between R and T site study fields were compared using unpaired *t*-test (GraphPad Prism version 6.04, GraphPad Software, La Jolla, California, USA).

## Results

### Mean density of OSR plants at study fields

OSR plant density at study fields of the R site varied between 18.3 plants/m² and 45.2 plants/m² (mean ± SD: 26.1 ± 7.2 plants/m²). Similarly, OSR plant density of the T site varied between 14.0 plants/m² and 49.6 plants/m² (mean ± SD: 31.1 ± 10.1 plants/m²) (Supplementary Figure S2A). There was no statistical difference between the mean plant densities of R and T site study fields (unpaired *t*-test: *t* = 1.684, *df* = 33, *P* = 0.102). Six study fields in the T site (T1, T9, T10, T13, T14 and T15) were drilled with more than one OSR variety (for more details, see: Heimbach et al. 2016). The mean number of OSR plants estimated for the different OSR varieties of these six study fields are illustrated in Supplementary Figure S2B. At the R site on average 68 % and at the T site on average 69 % of the drilled OSR seeds developed to OSR plants which survived the winter period of 2013/2014.

### Residues of clothianidin and metabolites—semi-field tunnel experiments

In all pollen samples collected by honey bees in tunnel tents(n = 34) from the 17 R site fields, residues of clothianidin, TZNG and TZMU were below the LOQ (*n* = 3) or even lower than the LOD (*n* = 31). In nectar samples no clothianidin, TZNG or TZMU could be detected (< LOD, *n* = 34) (Supplementary Table S1). TZMU and TZNG were not detected, neither in pollen nor in nectar samples (< LOD, one sample TZNG < LOQ) (Supplementary Table S1).

Clothianidin residue levels in all pollen samples collected by honey bees in tunnel tents from the 18 T site fields were on average 1.7 ± 0.8 μg/kg (mean ± SD; median: 1.6 μg/kg) in pollen and 1.3 ± 0.9 μg/kg (mean ± SD; median: 1.1 μg/kg) in nectar (*n* = 39 each) (Table1). In pollen samples, a maximum clothianidin concentration of 3.5 μg/kg was found, while nectar samples showed a maximum clothianidin concentration of 3.6 μg/kg (Supplementary Table S2). TZMU and TZNG were not detected in any of the 39 pollen and nectar samples from all study fields (< LOD) except for TZNG in 1 nectar sample and 9 pollen samples, where the concentration was below LOQ (Supplementary Table S2).

**Table 1:** Mean residue concentrations of clothianidin in pollen, nectar and honey sampled from bees both under semi-field and field realistic conditions

^a^ Minimum 90 % of samples with residues < LOD (= 0.3 µg/kg) or < LOQ (= 1.0 µg/kg), respectively

^b^ Calculation: residues < LOD = 0.0 µg/kg; residues < LOQ but >LOD = 0.65 µg/kg; residues > LOQ as quantified. See material and methods for details on calculation and text for standard deviations

The analysis of pollen and nectar samples from different study field subareas showed that concentrations of clothianidin could differ between subareas within the same field, even if only one OSR variety was cultivated there. For example, in nectar samples taken from two different subareas at study field T2, clothianidin was analysed in one subarea as < LOQ, whereas in another 3.6 µg/kg was detected. However, clothianidin residues in pollen samples from those two subareas at study field T2 were 1.6 and 1.1 µg/kg, respectively, and thus showed less variation (Supplementary Table S2).

### Residues of clothianidin and metabolites—field experiments (free flying bees)

#### Clothianidin residues in pollen, nectar and honey collected by honey bees

In all R site pollen samples collected by free flying honey bees from the first sampling date, residues of clothianidin, TZNG and TZMU were <LOD (*n* = 48). During the second sampling, residues of clothianidin were determined as < LOQ in one sample, whereas for all other samples (*n* = 47), clothianidin and its metabolites TZNG and TZMU were, again, determined as < LOD (Supplementary Table S3). At the T site, 12 out of 48 samples from the first sampling contained clothianidin concentrations < LOD, in 35 samples clothianidin residues were determined as < LOQ and one sample showed a quantifiable concentration of 1.1 µg/kg. At the second sampling, the clothianidin concentration increased slightly: concentrations remained < LOD in samples from 3 colonies, while 22 samples showed concentrations < LOQ and in 23 samples quantifiable concentrations of 1.0 – 2.7 µg/kg were analysed (Supplementary Table S4). For T site pollen samples, the average clothianidin concentration for the first sampling was 0.50 ± 0.30 µg/kg (mean ± SD; median: 0.65 µg/kg) and 0.96 ± 0.53 µg/kg (mean ± SD; median: 0.65 µg/kg) for the second sampling (Table 1). No measurable residues of the metabolites TZNG and TZMU were detected at both sampling dates (< LOD: *n* = 95; < LOQ: *n* = 1).

In nectar samples collected by free flying honey bees from both sampling dates at the R site, clothianidin residues were < LOD in 89 samples and < LOQ in 5 samples. Residues of TZNG and TZMU were < LOD in all samples analysed, except for two samples from the first sampling and one sample from the second sampling, where TZNG and TZMU were < LOQ (Supplementary Table S5). In nectar from both sampling dates at the T site, clothianidin concentrations were < LOD in 8 out of 96 samples and < LOQ in 66 out of 96 samples. Clothianidin was found in quantifiable concentrations in 22 out of 96 nectar samples, with 1.6 µg/kg being the maximum concentration found. The average clothianidin concentration for the first sampling was 0.67 ± 0.39 µg/kg (mean ± SD; median: 0.65 µg/kg) and for the second sampling 0.77 ± 0.24 µg/kg (mean ± SD; median: 0.65 µg/kg) (Table 1). Residues of TZMU were < LOQ in one sample and < LOD in the remaining 95 samples. Likewise, residues of TZNG were < LOD in all samples except for one, where it was < LOQ (Supplementary Table S6).

Honey samples were taken from each honey bee colony at the end of OSR flowering. Residues of clothianidin were < LOD in 18 out of 48 samples and < LOQ in 30 out of 48 samples from the R site. Residues of TZNG and TZMU were < LOD in all samples from the R site (Supplementary Table S7). In honey samples from the T site, 11 out of 48 samples showed clothianidin concentrations < LOQ. Hence, 37 out of 48 samples contained quantifiable concentrations of clothianidin ranging from 1.0 to 2.1 µg/kg. The average clothianidin concentration for all samples from the T site amounted to 1.35 ± 0.48 µg/kg (mean ± SD; median: 1.40 µg/kg) (Table 1). There were no differences between edge and distant study locations. Residues of TZNG as well as TZMU were <LOD in 34 samples and < LOQ in 14 samples (Supplementary Table S8).

#### Clothianidin residues in pollen collected by bumble bees

In total, 12 pollen samples were collected and analysed from returning bumble bee workers (one sample per study location). In all samples from the R site, concentrations of clothianidin, TZNG and TZMU were < LOD (Supplementary Table S9). Pollen from the T site contained clothianidin concentrations < LOQ in three samples, whereas the other three samples showed quantifiable concentrations with a maximum of 1.3 µg/kg (Supplementary Table S9). The average concentration of clothianidin in T site pollen samples was 0.88 ± 0.27 µg/kg (mean ± SD; median: 0.83 µg/kg) and thus was <LOQ (Table 1). The metabolites TZNG and TZMU could not be detected (< LOD) neither in R nor in T site samples, except for one sample from the T site, where TZNG was analysed as being < LOQ (Supplementary Table S9).

#### Clothianidin residues in pollen collected by mason bees

As for the sampling in bumble bees, 12 pollen samples were taken and analysed from provisioned mason bee cells (one pooled sample per study location). In all samples from the R site, concentrations of clothianidin, TZNG and TZMU were < LOD (Supplementary Table S10). Clothianidin concentrations in T site pollen samples were < LOQ in four samples, whereas the other two samples showed quantifiable concentrations with a maximum of 1.7 µg/kg (Supplementary Table S10). The average concentration of clothianidin in test site pollen samples was 0.88 ± 0.42 µg/kg (mean ± SD; median: 0.65 µg/kg) (Table 1). Both metabolites, TZNG and TZMU, could not be detected (< LOD) within samples from both the R and T sites (Supplementary Table S10).

## Discussion

Pollination by bees and insecticide treatments are essential components of modern agriculture. Unfortunately, potential bee toxicity of insecticides may cause problems for managed and wild bee populations (for recent reviews, see: Pisa et al. 2015; Goulson et al. 2015; Johnson 2015). Although the use of systemically acting neonicotinoid seed treatments is generally regarded a more ecologically sound alternative to foliar insecticide applications (Elbert et al. 2008), there is concern of pollinators be exposed to these chemical as they can be translocated into pollen and/or nectar when applied as seed treatments. However, potential negative effects of neonicotinoid seed-treatment of crops on pollinating insects critically depend on the concentrations to which they are exposed to by collecting pollen and nectar. Laboratory-based studies have clearly indicated adverse effects of neonicotinoids to bees at certain concentrations and under artificial exposure regimes (for reviews, see: Belzunces et al. 2012; Godfray et al. 2014; Godfray et al. 2015). However, no significant adverse effects have been detected in a number of field studies (Cutler and Scott-Dupree 2007, 2014; Blacquière et al. 2012; Pohorecka et al. 2012; Pilling et al. 2013; Cutler et al. 2014; Rolke et al. 2016; Sterk et al. 2016; Peters et al. 2016). This discrepancy maybe explained by pollinators experiencing lower PPP doses under field realistic conditions (Carreck and Ratnieks 2014). Therefore, data on neonicotinoid residues in pollen and nectar of neonicotinoid treated crops attractive to pollinating insects collected under common agricultural practice seem to be important for a realistic risk assessment.

This study presents data on residues of clothianidin and its active metabolites TZMU and TZNG in pollen, nectar and honey collected by bees in clothianidin seed-treated winter OSR. The OSR plants at all study fields developed relatively homogenously. Clothianidin, TZMU and TZNG were below LOD in almost all samples taken from the reference site, regardless of the sample matrix or collecting bee species. Thus, it can be assumed that honey bees, bumble bees and mason bees placed within the reference site exclusively foraged within this area free of clothianidin seed-treated OSR.

The species of honey bees, bumble bees and mason bees used in the present study are polylectic and thus collected pollen/nectar may become diluted by material from non-treated plants. Therefore, samples consisting exclusively of winter OSR pollen and nectar were analysed. This was achieved by forcing honey bees to collect pollen and nectar in tunnel tents. The resulting data provide information on the maximum possible concentrations when bees foraged on clothianidin-treated winter OSR only and thus are representative for a worst-case scenario. Average clothianidin concentrations of 1.7 ± 0.8 μg/kg in pollen and 1.3 ± 0.9 μg/kg in nectar were in the same range in both matrices. The maximal concentrations found in single samples were 3.5 µg/kg and 3.6 µg/kg in pollen and nectar, respectively. Thus, the concentrations were in the range of concentrations reported in previous field studies on OSR (Cutler and Scott-Dupree 2007; Cutler et al. 2014; Godfray et al. 2014; Godfray et al. 2015). Residues of TZMU and TZNG could not be detected or quantified in pollen and nectar samples from clothianidin seed-treated OSR even when collected in tunnel tents. This suggests that these two metabolites are only of minor importance for the risk assessment, at least for winter OSR in Central Europe.

Free flying honey bees, bumble bees and mason bees potentially had access to flowering plants other than OSR within the study sites (Heimbachet et al. 2016). Indeed, palynological analyses showed that the mean (±SD) percentage of OSR in pollen samples collected by honey bees at the test site ranged from 12.8 ± 13.8 % to 91.4 ± 2.7 % depending on study location and sampling date (Rolke et al. 2016). Similarly, the percentage of OSR in pollen sampled by bumble bees varied from 16 to 95 % (Sterk et al. 2016). Mason bees provisioned their brood cells with pollen consisting of 10.6 ± 6.8 % and 21.4 ± 13.2 % OSR pollen (Peters et al. 2016). OSR thus became the major pollen source, at least for honey bees and bumble bees during the course of our study (Rolke et al. 2016, Sterk et al. 2016). The percentage of OSR was lower than 100 % in almost all pollen samples taken from free flying bees (note that bumble bee-collected and mason bee-collected pollen samples for palynology and residue analysis were taken on different days). Due to dilution with pollen from other untreated plants, the concentrations of clothianidin have to be lower compared to those samples from the worst-case (semi-field) conditions. Indeed, average concentrations of clothianidin in honey bee-collected, bumble bee-collected and mason bee-collected pollen from the test site remain below LOQ (1.0 µg/kg).

This result is in accordance with other field studies. In a study conducted in Canada, on two experimental fields of spring OSR treated with PROSPER 8 FL and PONCHO 600 FS (delivering clothianidin at 4.0 g/kg seeds), for example, the majority of samples (>75 %) had no detectable level of clothianidin residues and the maximum concentration detected in pollen samples was 2.6 µg/kg (Cutler and Scott-Dupree 2007). In a more recent study, the same authors reported an average concentration of 0.8 ± 0.5 µg/kg in pollen samples collected by honey bees in PROSPER FX^®^-treated OSR (Cutler et al. 2014). This would fall below the LOQ when applying our method of residue analysis. Pohorecka et al. (2012) reported similar values for spring OSR treated with MODESTO 480 FS (4.9 g clothianidin/kg seeds) in a honey bee study conducted in Poland. Here, on average 0.6 ± 0.6 µg/kg (mean ± SD) and 2.2 ± 1.3 µg/kg (mean ± SD) clothianidin were detected in pollen loads and bee bread (processed pollen), respectively. In a German bee monitoring study, clothianidin was not detected in any of the 215 samples of honey bee bread collected from 2005–2007, although they included samples from colonies with high OSR input (Genersch et al. 2010).

Similar to residues in pollen, average concentrations of clothianidin in nectar samples taken from free flying honey bees were below the LOQ. Thus, the clothianidin residue levels remained slightly below the level that had been shown as worst-case scenario in tunnel tents. This indicates that free flying honey bees did not use OSR as an exclusive nectar source, but visited other plants for nectar foraging, as well. Since honey bees are polylectic, this is to be expected. The mixing of nectar from clothianidin seed-treated OSR and nectar from alternative non-treated plants explains the reduced clothianidin concentration found under realistic conditions. Nevertheless, OSR appears to be the major nectar source for honey bees, as has been shown by the palynological analysis of spring honey samples (Rolke et al. 2016). For test site colonies, the average percentage of OSR pollen in spring honey samples was 79.6 % ± 7.48 % (mean ± SD) and 77.9 % ± 8.93 % (mean ± SD) for edge and distant locations, respectively (Rolke et al. 2016). Thus, in the present study clothianidin concentrations in nectar samples were slightly lower than residue concentrations reported by Pohorecka et al. (2012), who analysed nectar samples from clothianidin seed-dressed spring OSR in Poland. These authors found 2.6 ± 4.0 µg/kg and 1.3 µg/kg depending on whether nectar was collected directly from rape flowers or nectar flow from combs was used, respectively (Pohorecka et al. 2012). In the Canadian study, Cutler et al. (2014) did not detect clothianidin residues in nectar collected by bees exposed to seed-treated OSR.

Bee larvae (and often adults) of eusocial bee species usually consume nectar in a more or less processed state. In honey bees, specialised workers process the collected nectar by adding glandular secretions and greatly reduce the water content to less than 20 % (Winston 1987). This modification and concentration leads to the production of honey. We analysed honey samples taken from colonies of free flying honey bees at the end of OSR flowering. The average clothianidin concentration was 1.35 ± 0.48 µg/kg in honey samples from test site colonies. Although mixing with nectar from plants other than OSR may have occurred (Rolke et al. 2016), clothianidin concentrations increased during the concentrating process of converting nectar into honey. However, clothianidin concentrations remained below LOQ in 23 % of the samples and the average concentration found was only slightly above LOQ (1.0 µg/kg). In general, clothianidin residues in honey stayed below the maximum residue level specified by the European Union (10 µg/kg, European Commission 2015). Clothianidin was not detected in honey samples in the Canadian study performed by Cutler et al. (2014), while in the Polish study slightly higher concentrations of clothianidin (3.4 ± 1.0 µg/kg) were found (Pohorecka et al. 2012).

In summary, the concentration of clothianidin in plant substrates collected by honey bees, bumble bees and mason bees under field conditions was found to be below 1.0 µg/kg (LOQ) in the majority of cases. Various studies have reported similar or slightly higher clothianidin concentrations in substrates originating from bees foraging near clothianidin seed-treated OSR fields (Cutler and Scott-Dupree 2007; Pohorecka et al. 2012; Pilling et al. 2013; Cutler et al. 2014). However, a recent study conducted by Rundlöf et al. (2015) in Sweden has shown considerably higher residual concentrations of clothianidin in pollen and nectar collected by honey bees and bumble bees in Elado^®^ seed-dressed spring OSR. These authors reported average clothianidin concentrations of 13.9 ± 1.8 µg/kg (mean ± SEM) and 10.3 ± 1.3 µg/kg (mean ± SEM) for honey bee-collected pollen and nectar, respectively, and 5.4 ± 1.4 µg/kg for bumble bee-collected nectar (Rundlöf et al., 2015). In comparison to our study, the Rundlöf et al. (2015) study clearly differed in at least two parameters. Firstly, spring OSR was planted in contrast to winter OSR in the present study. In the nectar (and pollen) samples from winter OSR fields, lower levels of neonicotinoid residues are usually found in comparison to spring OSR. This has, for example, been shown by Pohorecka et al. (2012) who found the concentration of thiamethoxam to be on average 1.6 µg/kg (in pollen < LOD) and 8.7 µg/kg (in pollen 5.6 µg/kg) in forage (nectar and honey) from winter OSR and spring OSR, respectively. These differences are most likely due to the longer period between planting neonicotinoid seed-dressed winter OSR and rape flowering (>6 months). During this time, partial degradation of neonicotinoids may occur. In contrast, only about 2 months usually pass from planting to flowering of spring OSR. Secondly, the seed density in the Rundlöf et al. (2015) study (7.7 kg OSR seeds/ha) was about twice as high as in the present study (3.4 ± 1.1 kg OSR seeds/ha in test fields; Heimbach et al. 2016). Together, these factors result in a considerably higher clothianidin load per ha and may have led to the higher residue values found in plant substrates in the Swedish study. In addition, the numbers of pollen and nectar samples analysed in the Rundlöf et al. (2015) study appear to be small (*n* = 5 honey bees per field for pollen sampling, *n* = 3–5 honey bees and bumble bees per field for nectar sampling). Due to high variation of residue concentrations in different parts of the same field, this may lead to a reduced discriminatory power. Nevertheless, the design of the present monitoring study followed the agricultural practice typical for winter OSR in the study area. Winter OSR is economically more important than spring OSR, e.g., the proportion of the cultivated area of winter OSR vs. spring OSR in the European Union in 2014 was 93.5 % and 99.7 % in Germany in 2015 (EUROSTAT 2016).

In conclusion, the residual concentrations of clothianidin and its active metabolites TZNG and TZMU in analysed samples of pollen, nectar and honey were clearly lower than the reported no observable adverse effect concentration for honey bees of 20 µg/kg, derived from feeding experiments using spiked diets (Schmuck and Keppler 2003). The clothianidin concentrations were also lower than those used in most laboratory-based studies that have reported adverse effects of clothianidin on bees (Godfray et al. 2014; Godfray et al. 2015; Thompson and Miles 2015). Accordingly, no detrimental effects on reproduction and health parameters on honey bees, bumble bees and mason bees exposed to clothianidin seed-treated OSR could be observed within the conditions of the present monitoring studies (Rolke et al. 2016; Sterk et al. 2016; Peters et al. 2016). Thus, it can be concluded that clothianidin seed-treated winter OSR, when used as directed, provides a favourable margin of safety for pollinating bees.

## Electronic supplementary material

Supplementary material
