# Supplementary material for: Large-scale monitoring of effects of clothianidin-dressed oilseed rape seeds on pollinating insects in northern Germany: residues of clothianidin in pollen, nectar and honey
Source: Ecotoxicology. 2016 Sep 20;25(9):1691–701. doi: 10.1007/s10646-016-1723-x (PMC5093202; doi:10.1007/s10646-016-1723-x)
Supplement: Supplementary file 1 — Supplementary material [file 10646_2016_1723_MOESM1_ESM.docx]

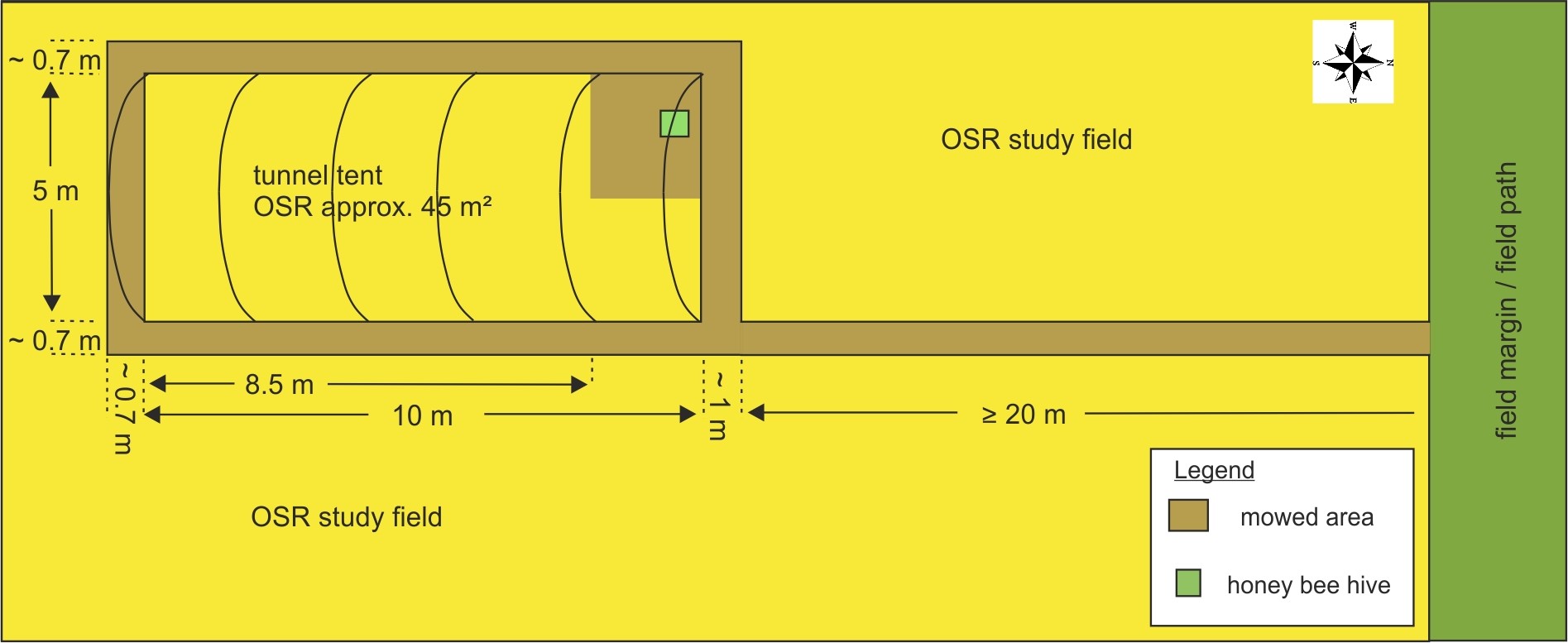


**Figure S1** Schematic drawing of tunnel tent setup inside a study field.


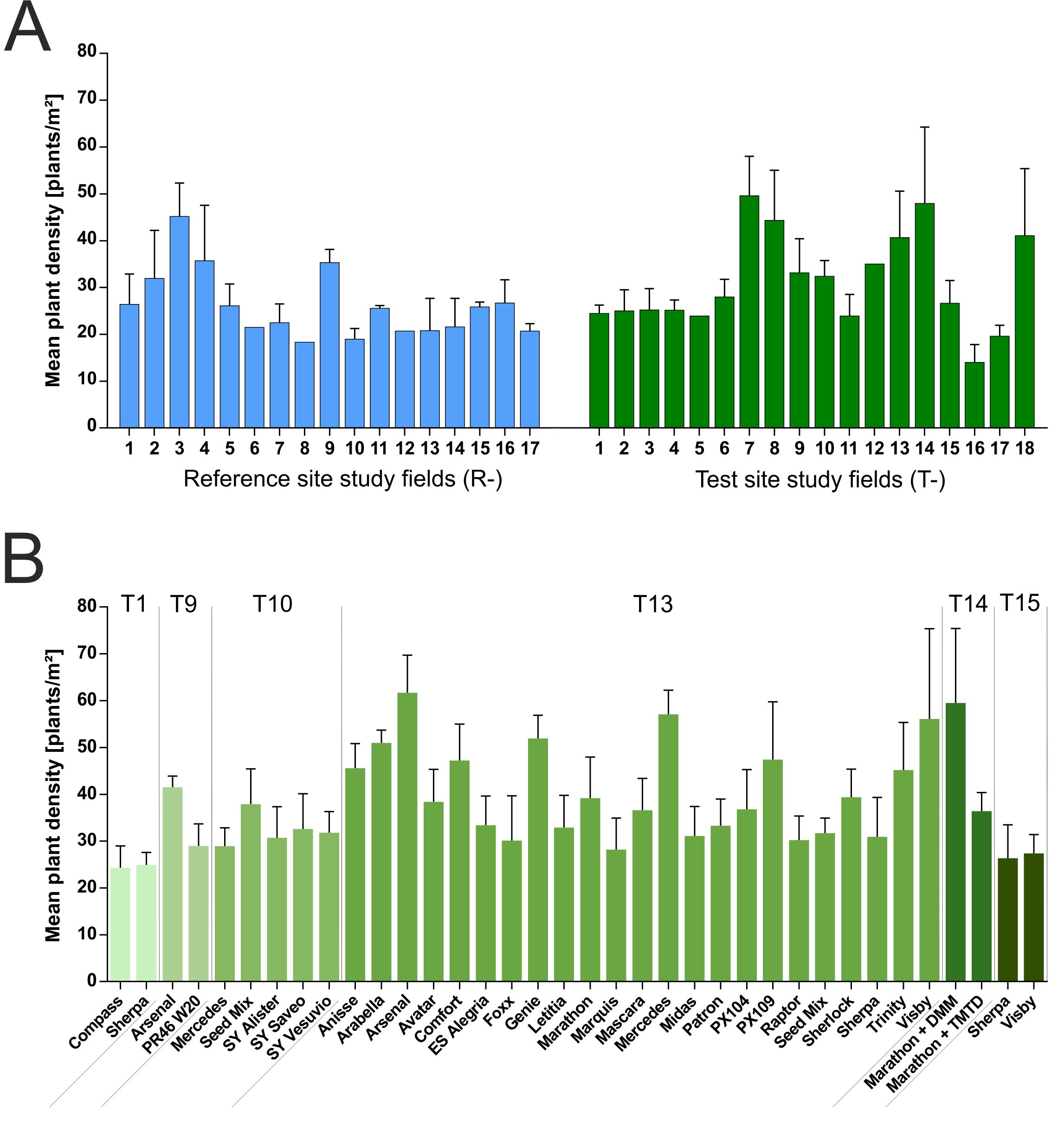


**Figure S2** Mean density of OSR plants of (A) the study fields of the reference and test site and of (B) the test site study fields with more than one mixed variety drilling.

**Table S1** Residue concentrations of clothianidin, thiazolylnitroguanidine (TZNG) and thiazolylmethylurea (TZMU) in nectar and pollen sampled from honey bees under semi-field conditions in tunnel tents at the reference site study field subareas. Values given in µg/kg. LOD = limit of detection (0.3 µg/kg), LOQ = limit of quantification (1.0 µg/kg).

**Table S2** Residue concentrations of clothianidin, thiazolylnitroguanidine (TZNG) and thiazolylmethylurea (TZMU) in nectar and pollen sampled from honey bees under semi-field conditions in tunnel tents at the treatment site study field subareas. Values given in µg/kg. LOD = limit of detection (0.3 µg/kg), LOQ = limit of quantification (1.0 µg/kg).

**Table S3** Residue concentrations of clothianidin, thiazolylnitroguanidine (TZNG) and thiazolylmethylurea (TZMU) in pollen sampled from honey bees under field conditions at the reference site. Values given in µg/kg. LOD = limit of detection (0.3 µg/kg), LOQ = limit of quantification (1.0 µg/kg).

| **Pollen collected by honey bees at the reference site** | | | | | | | | | | | |  |
| --- | --- | --- | --- | --- | --- | --- | --- | --- | --- | --- | --- | --- |
| **First Sampling** | | | | | | **Second Sampling** | | | | | |  |
| **Colony Code** | **Sample Date (DAP)** | | **Clothianidin in Pollen** | **TZNG in Pollen** | | **TZMU in Pollen** | **Sample Date (DAP)** | | **Clothianidin in Pollen** | **TZNG in Pollen** | **TZMU in Pollen** | |
|  |  |  | [µg/ kg] | [µg/ kg] | | [µg/ kg] |  |  | [µg/ kg] | [µg/ kg] | [µg/ kg] | |
| RA-1 | 15 | | < LOD | < LOD | | < LOD | 23 | | < LOD | < LOD | < LOD | |
| RA-2 | 15 | | < LOD | < LOD | | < LOD | 23 | | < LOD | < LOD | < LOD | |
| RA-3 | 15 | | < LOD | < LOD | | < LOD | 23 | | < LOD | < LOD | < LOD | |
| RA-4 | 15 | | < LOD | < LOD | | < LOD | 23 | | < LOD | < LOD | < LOD | |
| RA-5 | 15 | | < LOD | < LOD | | < LOD | 23 | | < LOD | < LOD | < LOD | |
| RA-6 | 15 | | < LOD | < LOD | | < LOD | 23 | | < LOD | < LOD | < LOD | |
| RA-7 | 15 | | < LOD | < LOD | | < LOD | 23 | | < LOD | < LOD | < LOD | |
| RA-8 | 15 | | < LOD | < LOD | | < LOD | 23 | | < LOD | < LOD | < LOD | |
| RB-1 | 15 | | < LOD | < LOD | | < LOD | 23 | | < LOD | < LOD | < LOD | |
| RB-2 | 15 | | < LOD | < LOD | | < LOD | 23 | | < LOD | < LOD | < LOD | |
| RB-3 | 15 | | < LOD | < LOD | | < LOD | 23 | | < LOD | < LOD | < LOD | |
| RB-4 | 15 | | < LOD | < LOD | | < LOD | 23 | | < LOD | < LOD | < LOD | |
| RB-5 | 15 | | < LOD | < LOD | | < LOD | 23 | | < LOD | < LOD | < LOD | |
| RB-6 | 15 | | < LOD | < LOD | | < LOD | 23 | | < LOD | < LOD | < LOD | |
| RB-7 | 15 | | < LOD | < LOD | | < LOD | 23 | | < LOD | < LOD | < LOD | |
| RB-8 | 15 | | < LOD | < LOD | | < LOD | 23 | | < LOD | < LOD | < LOD | |
| RC-1 | 15 | | < LOD | < LOD | | < LOD | 23 | | < LOQ | < LOD | < LOD | |
| RC-2 | 15 | | < LOD | < LOD | | < LOD | 23 | | < LOD | < LOD | < LOD | |
| RC-3 | 15 | | < LOD | < LOD | | < LOD | 23 | | < LOD | < LOD | < LOD | |
| RC-4 | 15 | | < LOD | < LOD | | < LOD | 23 | | < LOD | < LOD | < LOD | |
| RC-5 | 15 | | < LOD | < LOD | | < LOD | 23 | | < LOD | < LOD | < LOD | |
| RC-6 | 15 | | < LOD | < LOD | | < LOD | 23 | | < LOD | < LOD | < LOD | |
| RC-7 | 15 | | < LOD | < LOD | | < LOD | 23 | | < LOD | < LOD | < LOD | |
| RC-8 | 15 | | < LOD | < LOD | | < LOD | 23 | | < LOD | < LOD | < LOD | |
| RD-1 | 15 | | < LOD | < LOD | | < LOD | 19 | | < LOD | < LOD | < LOD | |
| RD-2 | 15 | | < LOD | < LOD | | < LOD | 19 | | < LOD | < LOD | < LOD | |
| RD-3 | 15 | | < LOD | < LOD | | < LOD | 23 | | < LOD | < LOD | < LOD | |
| RD-4 | 15 | | < LOD | < LOD | | < LOD | 23 | | < LOD | < LOD | < LOD | |
| RD-5 | 15 | | < LOD | < LOD | | < LOD | 23 | | < LOD | < LOD | < LOD | |
| RD-6 | 15 | | < LOD | < LOD | | < LOD | 23 | | < LOD | < LOD | < LOD | |
| RD-7 | 15 | | < LOD | < LOD | | < LOD | 23 | | < LOD | < LOD | < LOD | |
| RD-8 | 15 | | < LOD | < LOD | | < LOD | 23 | | < LOD | < LOD | < LOD | |
| RE-1 | 15 | | < LOD | < LOD | | < LOD | 23 | | < LOD | < LOD | < LOD | |
| RE-2 | 15 | | < LOD | < LOD | | < LOD | 23 | | < LOD | < LOD | < LOD | |
| RE-3 | 15 | | < LOD | < LOD | | < LOD | 23 | | < LOD | < LOD | < LOD | |
| RE-4 | 15 | | < LOD | < LOD | | < LOD | 23 | | < LOD | < LOD | < LOD | |
| RE-5 | 15 | | < LOD | < LOD | | < LOD | 23 | | < LOD | < LOD | < LOD | |
| RE-6 | 15 | | < LOD | < LOD | | < LOD | 23 | | < LOD | < LOD | < LOD | |
| RE-7 | 15 | | < LOD | < LOD | | < LOD | 23 | | < LOD | < LOD | < LOD | |
| RE-8 | 15 | | < LOD | < LOD | | < LOD | 23 | | < LOD | < LOD | < LOD | |
| RF-1 | 15 | | < LOD | < LOD | | < LOD | 23 | | < LOD | < LOD | < LOD | |
| RF-2 | 15 | | < LOD | < LOD | | < LOD | 23 | | < LOD | < LOD | < LOD | |
| RF-3 | 15 | | < LOD | < LOD | | < LOD | 23 | | < LOD | < LOD | < LOD | |
| RF-4 | 15 | | < LOD | < LOD | | < LOD | 23 | | < LOD | < LOD | < LOD | |
| RF-5 | 15 | | < LOD | < LOD | | < LOD | 23 | | < LOD | < LOD | < LOD | |
| RF-6 | 15 | | < LOD | < LOD | | < LOD | 23 | | < LOD | < LOD | < LOD | |
| RF-7 | 15 | | < LOD | < LOD | | < LOD | 23 | | < LOD | < LOD | < LOD | |
| RF-8 | 15 | | < LOD | < LOD | | < LOD | 23 | | < LOD | < LOD | < LOD | |
| **Mean^1)^** | | **< LOD** | | **< LOD** | **< LOD** | |  | **< LOD** | | **< LOD** | | **< LOD** |
| **Median^1)^** | | **< LOD** | | **< LOD** | **< LOD** | |  | **< LOD** | | **< LOD** | | **< LOD** |
| ^1)^ Mean and Median were calculated using 0 µg/kg for "< LOD" and 0.65 µg/kg²^)^ for "*< LOQ*"  ^2)^ Mean of LOD = 0.3 µg/ kg and LOQ = 1.0 µg/kg | | | | | | | | | | | | |

**Table S4** Residue concentrations of clothianidin, thiazolylnitroguanidine (TZNG) and thiazolylmethylurea (TZMU) in pollen sampled from honey bees under field conditions at the treatment site. Values given in µg/kg. LOD = limit of detection (0.3 µg/kg), LOQ = limit of quantification (1.0 µg/kg).

|  | **Pollen collected by honey bees at the test site** | | | | | | | |
| --- | --- | --- | --- | --- | --- | --- | --- | --- |
|  | **First sampling** | | | | **Second sampling** | | | |
| **Colony code** | **Sample date (DAP)** | **Clothianidin in pollen** [µg/ kg] | **TZNG in pollen** [µg/ kg] | **TZMU in pollen** [µg/ kg] | **Sample date (DAP)** | **Clothianidin in pollen** [µg/ kg] | **TZNG in**  **pollen** [µg/ kg] | **TZMU in pollen** [µg/ kg] |
|  |  |  |  |  |  |  |  |  |
| TA-1 | 15 | < LOQ | < LOD | < LOD | 23 | < LOQ | < LOD | < LOD |
| TA-2 | 15 | < LOQ | < LOD | < LOD | 23 | < LOQ | < LOD | < LOD |
| TA-3 | 15 | < LOQ | < LOD | < LOD | 23 | < LOQ | < LOD | < LOD |
| TA-4 | 15 | < LOD | < LOD | < LOD | 23 | < LOQ | < LOD | < LOD |
| TA-5 | 15 | < LOQ | < LOD | < LOD | 23 | 1.5 | < LOD | < LOD |
| TA-6 | 15 | < LOQ | < LOD | < LOD | 23 | < LOQ | < LOD | < LOD |
| TA-7 | 15 | < LOD | < LOD | < LOD | 23 | < LOQ | < LOD | < LOD |
| TA-8 | 15 | < LOQ | < LOD | < LOD | 23 | < LOQ | < LOD | < LOD |
| TB-1 | 15 | < LOQ | < LOD | < LOD | 19 | < LOQ | < LOD | < LOD |
| TB-2 | 15 | < LOQ | < LOD | < LOD | 19 | < LOQ | < LOD | < LOD |
| TB-3 | 15 | < LOQ | < LOD | < LOD | 19 | < LOQ | < LOD | < LOD |
| TB-4 | 15 | < LOQ | < LOD | < LOD | 19 | < LOQ | < LOD | < LOD |
| TB-5 | 15 | < LOQ | < LOD | < LOD | 19 | 1.1 | < LOD | < LOD |
| TB-6 | 15 | < LOQ | < LOD | < LOD | 19 | < LOQ | < LOD | < LOD |
| TB-7 | 15 | < LOQ | < LOD | < LOD | 19 | < LOQ | < LOD | < LOD |
| TB-8 | 15 | 1.1 | < LOD | < LOD | 19 | < LOQ | < LOD | < LOD |
| TC-1 | 15 | < LOQ | < LOD | < LOD | 23 | 1.2 | < LOD | < LOD |
| TC-2 | 15 | < LOQ | < LOD | < LOD | 23 | < LOQ | < LOD | < LOD |
| TC-3 | 15 | < LOQ | < LOD | < LOD | 23 | 1.0 | < LOD | < LOD |
| TC-4 | 15 | < LOQ | < LOD | < LOD | 23 | < LOQ | < LOD | < LOD |
| TC-5 | 15 | < LOQ | < LOD | < LOD | 23 | 1.1 | < LOD | < LOD |
| TC-6 | 15 | < LOQ | < LOD | < LOD | 23 | 1.0 | < LOD | < LOD |
| TC-7 | 15 | < LOQ | < LOD | < LOD | 23 | 1.0 | < LOD | < LOD |
| TC-8 | 15 | < LOQ | < LOD | < LOD | 23 | < LOQ | < LOD | < LOD |
| TD-1 | 15 | < LOQ | < LOD | < LOD | 23 | 1.4 | < LOD | < LOD |
| TD-2 | 15 | < LOD | < LOD | < LOD | 23 | 1.3 | < LOD | < LOD |
| TD-3 | 15 | < LOQ | < LOD | < LOD | 23 | 1.4 | < LOD | < LOD |
| TD-4 | 15 | < LOQ | < LOD | < LOD | 23 | 1.8 | < LOD | < LOD |
| TD-5 | 15 | < LOD | < LOD | < LOD | 23 | 2.3 | < LOD | < LOD |
| TD-6 | 15 | < LOQ | < LOQ | < LOQ | 23 | 1.5 | < LOD | < LOD |
| TD-7 | 15 | < LOQ | < LOD | < LOD | 23 | 1.6 | < LOD | < LOD |
| TD-8 | 15 | < LOQ | < LOD | < LOD | 23 | 1.3 | < LOD | < LOD |
| TE-1 | 15 | < LOD | < LOD | < LOD | 23 | < LOQ | < LOD | < LOD |
| TE-2 | 15 | < LOQ | < LOD | < LOD | 23 | < LOQ | < LOD | < LOD |
| TE-3 | 15 | < LOD | < LOD | < LOD | 23 | < LOQ | < LOD | < LOD |
| TE-4 | 15 | < LOD | < LOD | < LOD | 23 | 2.7 | < LOD | < LOD |
| TE-5 | 15 | < LOQ | < LOD | < LOD | 23 | < LOD | < LOD | < LOD |
| TE-6 | 15 | < LOQ | < LOD | < LOD | 23 | < LOD | < LOD | < LOD |
| TE-7 | 15 | < LOQ | < LOD | < LOD | 23 | < LOD | < LOD | < LOD |
| TE-8 | 15 | < LOQ | < LOD | < LOD | 23 | < LOQ | < LOD | < LOD |
| TF-1 | 15 | < LOQ | < LOD | < LOD | 23 | 1.7 | < LOD | < LOD |
| TF-2 | 15 | < LOD | < LOD | < LOD | 23 | 1.2 | < LOD | < LOD |
| TF-3 | 15 | < LOD | < LOD | < LOD | 23 | 1.6 | < LOD | < LOD |
| TF-4 | 15 | < LOQ | < LOD | < LOD | 23 | 1.1 | < LOD | < LOD |
| TF-5 | 15 | < LOD | < LOD | < LOD | 23 | 1.2 | < LOD | < LOD |
| TF-6 | 15 | < LOD | < LOD | < LOD | 23 | 1.1 | < LOD | < LOD |
| TF-7 | 15 | < LOQ | < LOD | < LOD | 23 | 1.0 | < LOD | < LOD |
| TF-8 | 15 | < LOD | < LOD | < LOD | 23 | < LOQ | < LOD | < LOD |
| **Mean^1)^** | | ***< LOQ* (0.50)** | **< LOD** | **< LOD** |  | ***< LOQ* (0.96)** | **< LOD** | **< LOD** |
| **Median^1)^** | | ***< LOQ* (0.65)** | **< LOD** | **< LOD** |  | ***< LOQ* (0.65)** | **< LOD** | **< LOD** |
| ^1)^ Mean and Median were calculated using 0.0 µg/kg for "< LOD" and 0.65^2)^ µg/kg for "*< LOQ*"  ^2)^ Mean of LOD = 0.3 µg/ kg and LOQ = 1.0 µg/kg | | | | | | | | |

**Table S5** Residue concentrations of clothianidin, thiazolylnitroguanidine (TZNG) and thiazolylmethylurea (TZMU) in nectar sampled from honey bees under field conditions at the reference site. Values given in µg/kg. LOD = limit of detection (0.3 µg/kg), LOQ = limit of quantification (1.0 µg/kg).

|  | **Nectar sampled by honey bees at the reference site** | | | | | | | |
| --- | --- | --- | --- | --- | --- | --- | --- | --- |
|  | **First sampling** | | | | **Second sampling** | | | |
| **Colony code** | **Sample date (DAP)** | **Clothianidin in nectar** | **TZNG in**  **nectar** | **TZMU in nectar** | **Sample date (DAP)** | **Clothianidin in nectar** | **TZNG in nectar** | **TZMU in nectar** |
|  |  | [µg/ kg] | [µg/ kg] | [µg/ kg] |  | [µg/ kg] | [µg/ kg] | [µg/ kg] |
| RA-1 | 10 | < LOD | < LOD | < LOD | 24 | < LOD | < LOD | < LOD |
| RA-2 | 10 | < LOD | < LOD | < LOD | 24 | < LOD | < LOD | < LOD |
| RA-3 | 10 | < LOD | < LOD | < LOD | 24 | < LOD | < LOD | < LOD |
| RA-4 | 10 | < LOD | < LOD | < LOD | 24 | < LOD | < LOD | < LOD |
| RA-5 | 10 | < LOD | < LOD | < LOD | 24 | < LOD | < LOD | < LOD |
| RA-6 | 10 | < LOD | < LOD | < LOD | 24 | < LOD | < LOD | < LOD |
| RA-7 | 10 | < LOD | < LOD | < LOD | 24 | < LOD | < LOD | < LOD |
| RA-8 | 10 | < LOD | < LOD | < LOD | 24 | < LOD | < LOD | < LOD |
| RB-1 | 15 | - | - | - | 24 | < LOD | < LOD | < LOD |
| RB-2 | 15 | < LOD | < LOD | < LOD | 24 | < LOQ | < LOD | < LOD |
| RB-3 | 15 | < LOD | < LOD | < LOD | 24 | < LOD | < LOD | < LOD |
| RB-4 | 15 | < LOD | < LOD | < LOD | 24 | < LOQ | < LOD | < LOD |
| RB-5 | 15 | < LOD | < LOD | < LOD | 24 | < LOD | < LOD | < LOD |
| RB-6 | 15 | < LOD | < LOD | < LOD | 24 | < LOD | < LOD | < LOD |
| RB-7 | 15 | < LOQ | < LOD | < LOD | 24 | < LOQ | < LOD | < LOD |
| RB-8 | 15 | < LOD | < LOD | < LOD | 24 | < LOQ | < LOD | < LOD |
| RC-1 | 14 | < LOD | < LOD | < LOD | 23 | < LOD | < LOD | < LOD |
| RC-2 | 14 | < LOD | < LOD | < LOD | 23 | < LOD | < LOD | < LOD |
| RC-3 | 14 | < LOD | < LOD | < LOD | 23 | < LOD | < LOD | < LOD |
| RC-4 | 14 | < LOD | < LOD | < LOD | 23 | < LOD | < LOD | < LOD |
| RC-5 | 14 | < LOD | < LOD | < LOD | 23 | < LOD | < LOD | < LOD |
| RC-6 | 14 | < LOD | < LOD | < LOD | 23 | < LOD | < LOD | < LOD |
| RC-7 | 14 | < LOD | < LOD | < LOD | 23 | < LOD | < LOD | < LOD |
| RC-8 | 14 | < LOD | < LOD | < LOD | 23 | < LOD | < LOD | < LOD |
| RD-1 | 14 | < LOD | < LOD | < LOD | 23 | < LOD | < LOD | < LOD |
| RD-2 | 14 | < LOD | < LOD | < LOD | 23 | < LOD | < LOD | < LOD |
| RD-3 | 14 | < LOD | < LOD | < LOD | 23 | < LOD | < LOD | < LOD |
| RD-4 | 14 | < LOD | < LOD | < LOD | 23 | < LOD | < LOD | < LOD |
| RD-5 | 14 | < LOD | < LOD | < LOD | 23 | < LOD | < LOD | < LOD |
| RD-6 | 14 | < LOD | < LOD | < LOD | 23 | < LOD | < LOD | < LOD |
| RD-7 | 14 | < LOD | < LOD | < LOD | 23 | < LOD | < LOD | < LOD |
| RD-8 | 14 | < LOD | < LOD | < LOD | 23 | < LOD | < LOD | < LOD |
| RE-1 | 15 | < LOD | < LOD | < LOD | 24 | < LOD | < LOD | < LOD |
| RE-2 | 15 | < LOD | < LOD | < LOD | 24 | < LOD | < LOD | < LOD |
| RE-3 | 15 | < LOD | < LOD | < LOD | 24 | < LOD | < LOD | < LOD |
| RE-4 | 15 | < LOD | < LOD | < LOD | 24 | < LOD | < LOD | < LOD |
| RE-5 | 15 | < LOD | < LOD | < LOD | 24 | < LOD | < LOD | < LOD |
| RE-6 | 15 | < LOD | < LOD | < LOD | 24 | < LOD | < LOD | < LOD |
| RE-7 | 15 | - | - | - | 24 | < LOD | < LOD | < LOD |
| RE-8 | 15 | < LOD | < LOD | < LOD | 24 | < LOD | < LOD | < LOD |
| RF-1 | 14 | < LOD | < LOD | < LOD | 23 | < LOD | < LOD | < LOD |
| RF-2 | 14 | < LOD | < LOD | < LOD | 23 | < LOD | < LOD | < LOD |
| RF-3 | 14 | < LOD | < LOD | < LOD | 23 | < LOD | < LOD | < LOD |
| RF-4 | 14 | < LOD | < LOD | < LOD | 23 | < LOD | < LOD | < LOD |
| RF-5 | 14 | < LOD | < LOD | < LOD | 23 | < LOD | < LOD | < LOD |
| RF-6 | 14 | < LOD | < LOD | < LOD | 23 | < LOD | < LOD | < LOD |
| RF-7 | 14 | < LOD | < LOD | < LOD | 23 | < LOD | < LOD | < LOD |
| RF-8 | 14 | < LOD | < LOD | < LOD | 23 | < LOD | < LOD | < LOD |
| **Mean^1)^** | | **< LOD** | **< LOD** | **< LOD** |  | ***<* LOD** | **< LOD** | **< LOD** |
| **Median^1)^** | | **< LOD** | **< LOD** | **< LOD** |  | **< LOD** | **< LOD** | **< LOD** |
| ^1)^ Mean and Median were RalRulated using 0.0 µg/kg for "< LOD" and 0.65 µg/kg^2)^ for "*< LOQ*"  ^2)^ Mean of LOD = 0.3 µg/ kg and LOQ = 1.0 µg/kg | | | | | | | | |

**Table S6** Residue concentrations of clothianidin, thiazolylnitroguanidine (TZNG) and thiazolylmethylurea (TZMU) in nectar sampled from honey bees under field conditions at the treatment site. Values given in µg/kg. LOD = limit of detection (0.3 µg/kg), LOQ = limit of quantification (1.0 µg/kg).

|  | **Nectar collected from honey bees at the test site** | | | | | | | |
| --- | --- | --- | --- | --- | --- | --- | --- | --- |
|  | **First sampling** | | | | **Second sampling** | | | |
| **Colony code** | **Sample date (DAP)** | **Clothianidin in nectar** | **TZNG in nectar** | **TZMU in nectar** | **Sample date (DAP)** | **Clothianidin in nectar** | **TZNG in nectar** | **TZMU in nectar** |
|  |  | [µg/ kg] | [µg/ kg] | [µg/ kg] |  | [µg/ kg] | [µg/ kg] | [µg/ kg] |
| TA-1 | 15 | LOD | < LOD | < LOD | 21 | LOQ | < LOD | < LOD |
| TA-2 | 15 | LOQ | < LOD | < LOD | 21 | LOQ | < LOD | < LOD |
| TA-3 | 15 | LOQ | < LOD | < LOD | 21 | LOQ | < LOD | < LOD |
| TA-4 | 15 | LOQ | < LOD | < LOD | 21 | LOQ | < LOD | < LOD |
| TA-5 | 15 | LOQ | < LOD | < LOD | 21 | LOQ | < LOD | < LOD |
| TA-6 | 15 | LOQ | < LOD | < LOD | 21 | LOQ | < LOD | < LOD |
| TA-7 | 15 | LOD | < LOD | < LOD | 21 | LOQ | < LOD | < LOD |
| TA-8 | 15 | LOQ | < LOD | < LOD | 21 | LOQ | < LOD | < LOD |
| TB-1 | 15 | 1.6 | < LOD | < LOD | 21 | LOQ | < LOD | < LOD |
| TB-2 | 15 | LOQ | < LOD | < LOD | 21 | LOQ | < LOD | < LOD |
| TB-3 | 15 | 1.3 | < LOD | < LOD | 21 | 1.0 | < LOD | < LOD |
| TB-4 | 15 | 1.2 | < LOD | < LOD | 21 | LOQ | < LOD | < LOD |
| TB-5 | 15 | LOQ | < LOD | < LOD | 21 | 1.3 | < LOD | < LOD |
| TB-6 | 15 | LOQ | < LOD | < LOD | 21 | 1.0 | < LOD | < LOD |
| TB-7 | 15 | LOQ | < LOD | < LOD | 21 | LOQ | < LOD | < LOD |
| TB-8 | 15 | 1.4 | < LOD | < LOD | 21 | LOQ | < LOD | < LOD |
| TC-1 | 16 | LOQ | < LOD | < LOD | 23 | LOQ | < LOD | < LOD |
| TC-2 | 16 | LOQ | < LOD | < LOD | 23 | LOQ | < LOD | < LOD |
| TC-3 | 16 | LOQ | < LOD | < LOD | 23 | LOQ | < LOD | < LOD |
| TC-4 | 16 | LOQ | < LOD | < LOD | 23 | LOQ | < LOD | < LOD |
| TC-5 | 16 | LOQ | < LOD | < LOD | 23 | LOQ | < LOD | < LOD |
| TC-6 | 16 | LOQ | < LOD | < LOD | 23 | LOQ | < LOD | < LOD |
| TC-7 | 16 | LOQ | < LOD | < LOD | 23 | 1.0 | < LOD | < LOD |
| TC-8 | 16 | LOQ | < LOD | < LOD | 23 | LOQ | < LOD | < LOD |
| TD-1 | 15 | LOQ | < LOD | < LOD | 23 | LOQ | < LOD | < LOD |
| TD-2 | 15 | LOQ | < LOD | < LOD | 23 | 1.5 | < LOD | < LOD |
| TD-3 | 15 | LOQ | < LOD | < LOD | 23 | 1.1 | < LOD | < LOD |
| TD-4 | 15 | LOQ | < LOD | < LOD | 23 | LOQ | < LOD | < LOD |
| TD-5 | 15 | LOQ | < LOD | < LOD | 23 | LOQ | < LOD | < LOD |
| TD-6 | 15 | 1.2 | < LOD | < LOD | 23 | 1.4 | < LOD | < LOD |
| TD-7 | 15 | 1.2 | < LOD | < LOD | 23 | LOQ | < LOD | < LOD |
| TD-8 | 15 | 1.3 | < LOD | < LOD | 23 | 1.3 | < LOD | < LOD |
| TE-1 | 15 | LOD | < LOD | < LOD | 23 | LOQ | < LOD | < LOD |
| TE-2 | 15 | LOD | < LOD | < LOD | 23 | LOQ | < LOD | < LOD |
| TE-3 | 15 | LOQ | < LOD | < LOD | 23 | LOQ | < LOD | < LOD |
| TE-4 | 15 | LOD | < LOD | < LOD | 23 | LOQ | < LOD | < LOD |
| TE-5 | 15 | LOD | < LOD | < LOD | 23 | 1.1 | LOQ | LOQ |
| TE-6 | 15 | LOD | < LOD | < LOD | 23 | LOQ | < LOD | < LOD |
| TE-7 | 15 | LOQ | < LOD | < LOD | 23 | LOQ | < LOD | < LOD |
| TE-8 | 15 | LOD | < LOD | < LOD | 23 | LOQ | < LOD | < LOD |
| TF-1 | 15 | 1.1 | < LOD | < LOD | 21 | LOQ | < LOD | < LOD |
| TF-2 | 15 | LOQ | < LOD | < LOD | 21 | LOQ | < LOD | < LOD |
| TF-3 | 15 | 1.0 | < LOD | < LOD | 21 | 1.4 | < LOD | < LOD |
| TF-4 | 15 | 1.2 | < LOD | < LOD | 21 | LOQ | < LOD | < LOD |
| TF-5 | 15 | LOQ | < LOD | < LOD | 21 | LOQ | < LOD | < LOD |
| TF-6 | 15 | LOQ | < LOD | < LOD | 21 | 1.0 | < LOD | < LOD |
| TF-7 | 15 | LOQ | < LOD | < LOD | 21 | LOQ | < LOD | < LOD |
| TF-8 | 15 | 1.1 | < LOD | < LOD | 21 | LOQ | < LOD | < LOD |
| **Mean^1)^** | | ***< LOQ* (0.67)** | **< LOD** | **< LOD** |  | ***< LOQ* (0.77)** | **< LOD** | **< LOD** |
| **Median^1)^** | | ***< LOQ* (0.65)** | **< LOD** | **< LOD** |  | ***< LOQ* (0.65)** | **< LOD** | **< LOD** |
| ^1)^ Mean and Median were calculated using 0.0 µg/kg for "< LOD" and 0.65 µg/kg^2)^ for "*< LOQ*"  ^2)^ Mean of LOD = 0.3 µg/ kg and LOQ = 1.0 µg/kg | | | | | | | | |

**Table S7** Residue concentrations of clothianidin, thiazolylnitroguanidine (TZNG) and thiazolylmethylurea (TZMU) in spring honey sampled from honey bee colonies under field conditions at the reference site. Values given in µg/kg. LOD = limit of detection (0.3 µg/kg), LOQ = limit of quantification (1.0 µg/kg).

| **Study location** | **Colony code** | **Sample date (DAP)** | **Clothianidin in honey**  [µg/ kg] | **TZNG in honey** [µg/ kg] | **TZMU in honey** [µg/ kg] |
| --- | --- | --- | --- | --- | --- |
| RA | RA-1 | 35 | < LOD | < LOD | < LOD |
| RA | RA-2 | 35 | < LOD | < LOD | < LOD |
| RA | RA-3 | 35 | < LOQ | < LOD | < LOD |
| RA | RA-4 | 35 | < LOQ | < LOD | < LOD |
| RA | RA-5 | 38 | < LOQ | < LOD | < LOD |
| RA | RA-6 | 38 | < LOQ | < LOD | < LOD |
| RA | RA-7 | 38 | < LOQ | < LOD | < LOD |
| RA | RA-8 | 38 | < LOQ | < LOD | < LOD |
| RB | RB-1 | 38 | < LOD | < LOD | < LOD |
| RB | RB-2 | 38 | < LOD | < LOD | < LOD |
| RB | RB-3 | 34 | < LOQ | < LOD | < LOD |
| RB | RB-4 | 34 | < LOD | < LOD | < LOD |
| RB | RB-5 | 34 | < LOQ | < LOD | < LOD |
| RB | RB-6 | 34 | < LOQ | < LOD | < LOD |
| RB | RB-7 | 34 | < LOQ | < LOD | < LOD |
| RB | RB-8 | 34 | < LOD | < LOD | < LOD |
| RC | RC-1 | 38 | < LOQ | < LOD | < LOD |
| RC | RC-2 | 38 | < LOQ | < LOD | < LOD |
| RC | RC-3 | 38 | < LOQ | < LOD | < LOD |
| RC | RC-4 | 38 | < LOQ | < LOD | < LOD |
| RC | RC-5 | 36 | < LOD | < LOD | < LOD |
| RC | RC-6 | 36 | < LOQ | < LOD | < LOD |
| RC | RC-7 | 36 | < LOD | < LOD | < LOD |
| RC | RC-8 | 36 | < LOQ | < LOD | < LOD |
| RD | RD-1 | 38 | < LOQ | < LOD | < LOD |
| RD | RD-2 | 35 | < LOD | < LOD | < LOD |
| RD | RD-3 | 35 | < LOQ | < LOD | < LOD |
| RD | RD-4 | 35 | < LOD | < LOD | < LOD |
| RD | RD-5 | 38 | < LOQ | < LOD | < LOD |
| RD | RD-6 | 35 | < LOQ | < LOD | < LOD |
| RD | RD-7 | 35 | < LOQ | < LOD | < LOD |
| RD | RD-8 | 35 | < LOQ | < LOD | < LOD |
| RE | RE-1 | 38 | < LOD | < LOD | < LOD |
| RE | RE-2 | 38 | < LOD | < LOD | < LOD |
| RE | RE-3 | 36 | < LOQ | < LOD | < LOD |
| RE | RE-4 | 38 | < LOQ | < LOD | < LOD |
| RE | RE-5 | 38 | < LOD | < LOD | < LOD |
| RE | RE-6 | 35 | < LOD | < LOD | < LOD |
| RE | RE-7 | 35 | < LOQ | < LOD | < LOD |
| RE | RE-8 | 36 | < LOQ | < LOD | < LOD |
| RF | RF-1 | 36 | < LOQ | < LOD | < LOD |
| RF | RF-2 | 36 | < LOQ | < LOD | < LOD |
| RF | RF-3 | 36 | < LOQ | < LOD | < LOD |
| RF | RF-4 | 38 | < LOD | < LOD | < LOD |
| RF | RF-5 | 36 | < LOD | < LOD | < LOD |
| RF | RF-6 | 36 | < LOD | < LOD | < LOD |
| RF | RF-7 | 36 | < LOD | < LOD | < LOD |
| RF | RF-8 | 38 | < LOQ | < LOD | < LOD |
| **Mean^1)^** | | | ***< LOQ* (0.41)** | **< LOD** | **< LOD** |
| **Median^1)^** | | | ***< LOQ* (0.65)** | **< LOD** | **< LOD** |

^1)^ Mean and Median were calculated using 0.0 µg/kg for "< LOD" and 0.65 µg/kg^2)^ for "*< LOQ*"

^2)^ Mean of LOD = 0.3 µg/ kg and LOQ = 1.0 µg/kg

**Table S8** Residue concentrations of clothianidin, thiazolylnitroguanidine (TZNG) and thiazolylmethylurea (TZMU) in spring honey sampled from honey bee colonies under field conditions at the treatment site. Values given in µg/kg. LOD = limit of detection (0.3 µg/kg), LOQ = limit of quantification (1.0 µg/kg).

| **Study location** | **Colony code** | **Sample date**  **(DAP)** | **Clothianidin in honey**  [µg/ kg] | **TZNG in honey** [µg/ kg] | **TZMU in honey** [µg/ kg] |
| --- | --- | --- | --- | --- | --- |
| TA | TA-1 | 38 | 1.0 | < LOD | < LOD |
| TA | TA-2 | 38 | < LOQ | < LOD | < LOD |
| TA | TA-3 | 38 | < LOQ | < LOD | < LOD |
| TA | TA-4 | 38 | < LOQ | < LOD | < LOD |
| TA | TA-5 | 37 | 1.4 | < LOD | < LOD |
| TA | TA-6 | 37 | 1.0 | < LOD | < LOD |
| TA | TA-7 | 37 | < LOQ | < LOD | < LOD |
| TA | TA-8 | 37 | < LOQ | < LOD | < LOD |
| TB | TB-1 | 38 | 1.5 | < LOD | < LOD |
| TB | TB-2 | 38 | 1.2 | < LOD | < LOD |
| TB | TB-3 | 38 | 1.6 | < LOD | < LOD |
| TB | TB-4 | 35 | 1.5 | < LOD | < LOD |
| TB | TB-5 | 35 | 1.3 | < LOD | < LOD |
| TB | TB-6 | 35 | 1.3 | < LOD | < LOD |
| TB | TB-7 | 35 | 1.2 | < LOQ | < LOQ |
| TB | TB-8 | 34 | 1.3 | < LOD | < LOD |
| TC | TC-1 | 37 | 1.7 | < LOQ | < LOQ |
| TC | TC-2 | 37 | 1.5 | < LOQ | < LOQ |
| TC | TC-3 | 37 | 1.8 | < LOQ | < LOQ |
| TC | TC-4 | 38 | 2.1 | < LOQ | < LOQ |
| TC | TC-5 | 37 | 1.9 | < LOQ | < LOQ |
| TC | TC-6 | 37 | 1.9 | < LOD | < LOD |
| TC | TC-7 | 38 | 1.9 | < LOQ | < LOQ |
| TC | TC-8 | 35 | 1.8 | < LOQ | < LOQ |
| TD | TD-1 | 34 | 1.4 | < LOD | < LOD |
| TD | TD-2 | 34 | 1.6 | < LOD | < LOD |
| TD | TD-3 | 34 | 1.2 | < LOD | < LOD |
| TD | TD-4 | 34 | 1.2 | < LOD | < LOD |
| TD | TD-5 | 38 | 1.1 | < LOD | < LOD |
| TD | TD-6 | 38 | 1.5 | < LOD | < LOD |
| TD | TD-7 | 38 | 1.7 | < LOD | < LOD |
| TD | TD-8 | 38 | 1.8 | < LOQ | < LOQ |
| TE | TE-1 | 35 | 1.3 | < LOD | < LOD |
| TE | TE-2 | 35 | 1.0 | < LOD | < LOD |
| TE | TE-3 | 35 | < LOQ | < LOD | < LOD |
| TE | TE-4 | 37 | < LOQ | < LOD | < LOD |
| TE | TE-5 | 37 | < LOQ | < LOD | < LOD |
| TE | TE-6 | 37 | < LOQ | < LOD | < LOD |
| TE | TE-7 | 37 | < LOQ | < LOD | < LOD |
| TE | TE-8 | 38 | < LOQ | < LOD | < LOD |
| TF | TF-1 | 38 | 2.0 | < LOQ | < LOQ |
| TF | TF-2 | 34 | 1.9 | < LOQ | < LOQ |
| TF | TF-3 | 35 | 1.7 | < LOD | < LOD |
| TF | TF-4 | 35 | 2.0 | < LOQ | < LOQ |
| TF | TF-5 | 35 | 2.0 | < LOD | < LOD |
| TF | TF-6 | 38 | 1.8 | < LOQ | < LOQ |
| TF | TF-7 | 35 | 2.0 | < LOQ | < LOQ |
| TF | TF-8 | 38 | 1.7 | < LOD | < LOD |
| **Mean^1)^** | | | **1.35** | **< LOD** | **< LOD** |
| **Median^1)^** | | | **1.40** | **< LOD** | **< LOD** |

^1)^ Mean and Median were calculated using 0.0 µg/kg for "< LOD" and 0.65 µg/kg^2)^ for "*< LOQ*"

^2)^ Mean of LOD = 0.3 µg/ kg and LOQ = 1.0 µg/kg

**Table S9** Residue concentrations of clothianidin, thiazolylnitroguanidine (TZNG) and thiazolylmethylurea (TZMU) in pollen sampled from bumble bees under field conditions at both the reference (R) and treatment (T) site. Values given in µg/kg. LOD = limit of detection (0.3 µg/kg), LOQ = limit of quantification (1.0 µg/kg).

| **Study location** | **Pollen collected by bumble bees** | | |
| --- | --- | --- | --- |
|  | **Clothianidin**  [µg/kg] | **TZNG**  [µg/kg] | **TZMU**  [µg/kg] |
| RA | < LOD | < LOD | < LOD |
| RB | < LOD | < LOD | < LOD |
| RC | < LOD | < LOD | < LOD |
| RD | < LOD | < LOD | < LOD |
| RE | < LOD | < LOD | < LOD |
| RF | < LOD | < LOD | < LOD |
| TA | < LOQ | < LOD | < LOD |
| TB | < LOQ | < LOD | < LOD |
| TC | 1.0 | < LOQ | < LOD |
| TD | 1.0 | < LOD | < LOD |
| TE | < LOQ | < LOD | < LOD |
| TF | 1.3 | < LOD | < LOD |
| LOD = 0.3 µg/kg, LOQ = 1.0 µg/kg | | | |

**Table S10** Residue concentrations of clothianidin, thiazolylnitroguanidine (TZNG) and thiazolylmethylurea (TZMU) in pollen sampled from mason bees under field conditions at both the reference (R) and treatment (T) site. Values given in µg/kg. LOD = limit of detection (0.3 µg/kg), LOQ = limit of quantification (1.0 µg/kg).

| **Study location** | **Pollen collected by mason bees** | | |
| --- | --- | --- | --- |
|  | **Clothianidin**  [µg/kg] | **TZNG**  [µg/kg] | **TZMU**  [µg/kg] |
| ORA | < LOD | < LOD | < LOD |
| ORB | < LOD | < LOD | < LOD |
| ORC | < LOD | < LOD | < LOD |
| ORD | < LOD | < LOD | < LOD |
| ORE | < LOD | < LOD | < LOD |
| ORF | < LOD | < LOD | < LOD |
| OTA | < LOQ | < LOD | < LOD |
| OTB | 1.1 | < LOD | < LOD |
| OTC | 1.7 | < LOD | < LOD |
| OTD | < LOQ | < LOD | < LOD |
| OTE | < LOQ | < LOD | < LOD |
| OTF | < LOQ | < LOD | < LOD |
| LOD = 0.3 µg/kg, LOQ = 1.0 µg/kg | |  |  |
|  | | | |
